# Recent Progress in the Management of Obesity

**DOI:** 10.3390/nu15122651

**Published:** 2023-06-06

**Authors:** Javier Gómez-Ambrosi

**Affiliations:** 1 Metabolic Research Laboratory, Clínica Universidad de Navarra, 31008 Pamplona, Spain; jagomez@unav.es; 2 Centro de Investigación Biomédica en Red-Fisiopatología de la Obesidad y Nutrición (CIBEROBN), Instituto de Salud Carlos III, 31008 Pamplona, Spain; 3 Obesity and Adipobiology Group, Instituto de Investigación Sanitaria de Navarra (IdiSNA), 31008 Pamplona, Spain

Obesity represents the most prevalent metabolic disease nowadays, posing a significant public health risk. This situation has led to a better understanding of the systems that regulate body weight and energy homeostasis. Obesity shortens life expectancy by increasing the risk of developing comorbidities such as type 2 diabetes (T2D), cardiovascular disease, fatty liver disease, and several types of cancer, among other conditions [1]. Reduced calorie intake and increased energy expenditure have traditionally been the cornerstones of the therapeutic strategy for patients living with obesity. Obesity-related comorbidities can significantly improve even with a small amount of weight loss [2]. This Special Issue includes some of the most notable progress achieved in recent years in the treatment of patients with obesity.

A better understanding of the ethiopathology of obesity should represent the pillar on which to base a good management for this condition. In this sense, in recent years, we have expanded our knowledge about the wide array of drivers that can facilitate or contribute to the development of obesity. Compiling most of these factors, the review by Catalán et al. summarizes many of the obesogens that may explain the increasing prevalence of obesity worldwide [3]. Besides “classical” direct causes, such as genetic and behavioral determinants of energy intake and expenditure, the review includes some less appreciated drivers of the excess adiposity epidemic, such as the microbiota, infectobesity, the influence of chronobiology, and the roles of endocrine disrupters, urban planning and climate change. Their review evidences the relevance of the “exposome” in the development and perpetuation of the obesity epidemic [3]. Archer and Lavie bring an interesting perspective according to which effective management strategies need a personalized approach that takes into account the subtyping of obesity phenotypes. They distinguish between acquired and inherited obesity. The former refers to the development of excessive adiposity after puberty; because acquired obesity is behavioral in origin, it can be responsive to dietary and exercise-based therapies. On the other hand, inherited obesity includes all types of obesities that occur before pubescence (infancy and childhood) and are present at birth, which would be less susceptible to treatment [4]. Having accessible tools that allow us to properly phenotype patients with obesity considering their cardiometabolic risk is essential to establish the most appropriate treatment [1]. In this sense, Sanchez et al. [5] describe the use of the measurement of skin autofluorescence (SAF), a non-invasive estimator of advanced glycation endproducts (AGEs), in patients with obesity. Although SAF correlates with body fat percentage estimated with the CUN-BAE [6], it is not increased in individuals with obesity, being more related to the presence of cardiometabolic risk factors. The authors suggest that SAF may represent a useful tool for the identification of individuals with unhealthy obesity, opening the door to new approaches to managing obesity in clinical practice [5].

A change in dietary habits is still the first step in the treatment of obesity. With a focus on components of the Mediterranean diet (MD) that may help to maintain proper mitochondrial function, Portincasa’s group extensively reviews the benefits of this diet, providing cellular and animal models as well as clinical trials in individuals with metabolic syndrome assessing the efficacy of MD components on mitochondrial structure and activity [7]. On the other hand, a high intake of ultra-processed food (UPF) has been related to an increased risk of obesity and obesity-associated comorbidities. It has been debated for a long time whether ultra-processing itself is harmful or if UPFs just have a reduced nutritional content. Dicken and Batterham, in an exhaustive review, demonstrate that, consistently across different studies, adjustment for fat, sugar and sodium intake, or for adherence to a variety of healthy or unhealthy dietary patterns, has a very limited impact on the detrimental relationship between UPF intake and a diverse range of health-related outcomes [8]. These findings cast doubt on the claim that the negative effects of UPFs can be entirely attributable to their nutritional content and clearly suggest that features of ultra-processing are significant determinants that have an impact on obesity and on health in general [8].

Phase angle (PA) could be used as marker of health status in relation to nutrition, including in patients with obesity, to monitor the efficacy of weight loss and skeletal muscle mass preservation [9]. Basiri and colleagues show in this Special Issue that a treatment with nutritional supplements and diet education in addition to the standard care in patients with overweight or obesity and diabetic foot ulcers has positive ponderal and metabolic effects, including a tendency towards a lower decrease in PA. Given that an increase in PA is associated with a reduction in the risk of mortality in patients with diabetes, their findings may be considered clinically relevant [10].

Having tools capable of reliably predicting weight loss throughout a nutritional intervention has been shown to be very useful during dietary treatment in patients with overweight or obesity. Markovikj et al. report that a modification of the Wishnofsky equation, described several decades ago to determine the body mass loss in a dietary intervention based on the timeframe of energy intake reduction, accurately predicts weight loss in 100 adults with overweight or obesity under a ketogenic diet [11].

When lifestyle modification fails, and before considering bariatric surgery, pharmacological interventions should be considered as an alternative therapy for weight loss. In this sense, achieving a normal weight via long-term drug therapy with appropriate tolerability and safety has remained a difficult challenge until recently. However, in recent years, new drugs or combinations of thereof, for example semaglutide and tirzepatide, providing mean weight loss well above 10% and improving cardiovascular outcomes in patients with T2D give hope for the future [12]. The scoping review by le Roux’s group reports that the results of the Semaglutide Treatment Effect in People with Obesity (STEP) trials confirm the efficacy of once weekly 2.4 mg semaglutide on weight loss in patients with obesity [13]. Although semaglutide produced some gastrointestinal-related side effects, it was in general safe and well tolerated. Given the effectiveness of the drug, the authors wonder if nutritional therapy may have to be redefined and indicated to achieve better health instead for weight loss [13]. An original study included in the Special Issue carried out by the same group tried to delve into the mechanisms by which the duodenal-jejunal bypass liner (endobarrier) induces more pronounced weight loss than a conventional dietary treatment in patients with obesity and T2D. They conclude that the greater weight loss was due to mechanisms other than a reduction in energy intake or a change in food preferences [14].

The outbreak of the COVID-19 pandemic and the lockdown that accompanied it had a very notable impact on our lives, as well as on our health [15]. Due to the lockdown, health providers were forced to increase the use of telehealth and telemedicine. Gilardini and colleagues investigated the interest of patients with obesity in taking part in a remotely delivered multidisciplinary program for weight loss [16]. According to their findings, males and elder people were more reluctant than females and younger people to be involved in an online nutritional intervention. They also conclude that the use of telemedicine in the management of obesity could reduce lost workdays and patient travel time, increasing the number of subjects who could receive treatment and improving treatment adherence [16].

Finally, Abeltino et al. describe the usefulness of Personalized Metabolic Avatar (PMA) to predict the response to a diet [17]. By means of deep learning, they develop a data-driven metabolic model, derived from the information provided by smart bands and impedance balances, which allows simulations of diet programs, allowing the setting of customized targets for obtaining optimal weight [17,18].

We have witnessed progress in the treatment of obesity in recent years with, for example, the advances mentioned above. However, much remains to be done and further research must be carried out to improve and optimize the management of patients with obesity and to increase their quality of life.
